# Redox unbalance modifies neurogenic potential

**DOI:** 10.18632/oncotarget.14110

**Published:** 2016-12-22

**Authors:** Marilena Raciti, Sandra Ceccatelli

**Affiliations:** Department of Neuroscience, Karolinska Institutet, Stockholm, Sweden

**Keywords:** redox unbalance, neuronal differentiation, glucocorticoids, antioxidant

Reactive oxygen species (ROS), including superoxide anion, hydroxyl radicals and hydrogen peroxide, can be formed endogenously by incomplete reduction of molecular oxygen occurring during mitochondrial oxidative phosphorylation, or by interactions with exogenous sources. The antioxidant defense system, with its major enzymes Catalase, superoxide dismutase (SODs) and glutathione peroxidase (Gpx), has a critical role in counteracting the accumulation of ROS. An imbalance in ROS production and antioxidant capacity leads to oxidative stress. Several pathological conditions, including neurological and neuropsychiatric disorders, such as Parkinson’s disease, amyotrophic lateral sclerosis, autism, but also depression, have been associated to the occurrence of oxidative stress.

Besides the critical role played in diseases, the cellular redox state influences cell functions and viability. In fact, the modulation of ROS levels represents a major strategy to regulate gene expression, cell proliferation and differentiation already from early development [1]. This is related to the redox-responsiveness of several transcription factors (TFs), whose capability to bind DNA is modulated by the oxidation/reduction of critical cysteine residues contained in their DNA binding site [2]. In addition to TFs, the regulation of key signaling pathway requires the generation of ROS that activate essential proteins through the reversible oxidation of key cysteine residues [3].

Increasing evidence points to the critical role played by the ROS-mediated signaling in the regulation of neurogenesis [3], as low non-toxic ROS levels promote self-renewal and regulate the neurogenic potential in neural stem cells [3]. Therefore, misfunction of the antioxidant system and uncontrolled ROS generation may have detrimental consequences for the developing nervous system and even result in an increased predisposition to neurological and psychiatric disorders.

Early insults altering the redox state, such as certain xenobiotics contaminating food [4], can lead to long-lasting changes in the structure and function of the developing brain. Another adverse condition altering the ROS balance and leading to long-term pathological outcomes is the developmental exposure to high levels of glucocorticoids (GCs). GCs are steroid hormones critical for the differentiation of many fetal tissues as well as for the physiological response to stress. During pregnancy, the placental 11-β-hydroxysteroid dehydrogenase keeps fetal levels of GCs lower than maternal levels. However, prenatal exposure to excess GCs occurring in case of severe maternal stress, placenta failure or therapeutic treatment, has been shown to modify the development of the nervous system with life-long adverse effects.

Human induced pluripotent stem cells (IPSCs) have been proposed as a powerful *in vitro* model to investigate the molecular basis of neurodevelopment in normal and pathological conditions. We recently studied the long-lasting effects of the synthetic GC analog dexamethasone (Dex) on ROS balance and its impact on neuronal differentiation of human long-term self-renewing neuroepithelial-like stem cells (lt-NES cells), a relatively new IPSCs-derived *in vitro* model [5].

We observed Dex-induced heritable changes in the antioxidant system and ROS balance that were associated with altered differentiation potential of lt- NES. The increased intracellular ROS modified the normal commitment of neural progenitor cells, leading to a shift toward the glial fate at the expense of neuronal differentiation. Indeed, after Dex exposure the neuronal cell population was significantly reduced, as shown by a decreased number of TUJ1 expressing cells and a downregulation of specific neuronal markers, such as *MAP2*, *DCX*, *vGLUT2* and *GAD67*. Concomitantly, the glial marker *GFAP* was upregulated, while the expression of *PAX6* and *SOX2*, two key well-established neural progenitor cell markers, was only slightly increased, supporting the idea that Dex directs neural progenitor cells toward the glial fate rather than stalling differentiation [5]. Interestingly, there was a cause-effect relationship between the Dex-induced ROS unbalance and the differentiation impairment, as upon treatment with the well-established antioxidant molecule *N*-acetyl-cysteine (NAC), a normal expression pattern of both neuronal and glial markers could be restored [5].

The implications of these data are relevant for both the developing and the adult nervous system, as neurogenic niches are present also in the adult brain where they are crucially involved in memory, learning and response to injuries [6]. Consequently, the Dex-induced alterations we observed in lt-NES strongly support the hypothesis that early insults may result in long-term pathological outcomes in adulthood. This idea is further reinforced by our *in vivo* studies showing that prenatal exposure to high GCs concentration is associated with impaired hippocampal neurogenesis and depression-like behavior in adult mice [7].

As mentioned before, alterations in the redox state have been linked to the pathophysiology of neuropsychiatric disorders, such as depression, and antioxidants have been shown to improve the effects of antidepressants by replenishing glutathione [8].

Altogether, our findings support the so-called “neurogenic theory of depression”, proposing that major depressive disorder is associated with defective adult neurogenesis and that it is possible to recover from the disease by re-establishing a normal neurogenesis pattern.

The positive effect of NAC on Dex-exposed lt- NES shows the central role played by ROS in neuronal differentiation and reinforces the notion that treatment with antioxidants could be a valid complementary therapy for neuropsychiatric disorders that have been linked to impaired neurogenesis.

## References

[1] Tsatmali M (2006). Mol Cell Neurosci.

[2] Liu H (2005).

[3] Le Belle JE (2011). Cell Stem Cell.

[4] Onishchenko N (2008). J Neurochem.

[5] Raciti M (2016). Neuropharmacology.

[6] Delgado-Garcia LM (2016). Int J Stem Cell Res Ther Int Libr Cit Int J Stem Cell Res Ther Int J Stem
Cell Res Ther.

[7] Spulber S (2015). Transl Psychiatry.

[8] Maes M (2011). Prog Neuropsychopharmacol Biol Psychiatry.

